# The Prevalence of Psychiatric Disorders in Children and Adolescents in Hamadan Province, West of Iran

**Published:** 2018-12-10

**Authors:** Mohammad Ahmadpanah, Marzieh Nazaribadie, Mohammad Reza Mohammadi, Zahra Hooshyari, Seyyed Salman Alavi, Ali Ghaleiha, Leila Jahangard, Amir Keshavarzi, Majid Farahmand Sabet, Safdar Nabizadeh, Nafiseh Bagheri, Parvaneh Zaeri Omid, Jale Arji, Faeze Kokabi Heidarpoor, Donya Jedi Ghader, Saba Abbasi, Narges Toluei

**Affiliations:** ^1^ Research Center for Behavioral Disorders and Substance Abuse, Hamadan University of Medical Sciences, Hamadan, Iran; ^2^ Psychiatry and Psychology Research Center, Roozbeh Hospital, Tehran University of Medical Sciences, Tehran, Iran; ^3^ Department of Psychology of Razi University, Kermanshah, Iran; ^4^ Department of Psychology Psychology, Bu-Ali Sina University, Hamadan, Iran; ^5^ Department of Psychology, Islamic Azad University, Hamadan, Iran; ^6^ Department an of education sciences, Islamic Azad University, Shiraz Branch, Shiraz. Iran

**Keywords:** Prevalence, Mental disorders, Child and adolescent, Iran

## Abstract

**Background:** There are numerous reports regarding increasing childhood and adolescent mental health problems. The aim of this study was to determine the prevalence of psychiatric disorders in Hamadan Province, west of Iran from July 2016 to May 2017.

**Study design:** A cross-sectional study.

**Methods:** The sample included 1025 Hamadan residents selected using multistage cluster sampling. Psychiatric disorders were assessed by semi-structured psychiatric interview Kiddie-Sads-Present and Lifetime Version (K-SADS-PL). The data were analyzed using the SPSS software. We used the multivariable logistic regression to predict the Odds Ratios (ORs).

**Results:** The prevalence of total psychiatric disorder was 8.6%. Psychiatric disorders in boys were higher than girls (12.6% and 4.9%, respectively). The psychiatric disorders were most prevalent in 6-9 yr old age group (11%). The prevalence of behavioral disorder was 3.8% with attention deficit hyperactivity disorder (ADHD) as the most prevalent case (2.0%). The prevalence of anxiety disorder was 2.8% in which the highest prevalence belonged to separation anxiety disorder (SAD) (1.1%). The prevalence of neurodevelopment disorder was 1.5% with the highest prevalence of 1% observed in epilepsy. The prevalence of mood disorder was 1.1% with the depressive disorder as the most prevalent one (1.0%). The prevalence of enuresis was 2.7%. The most common comorbidities were anxiety and mood disorders 5(50.0%).

**Conclusion:** The prevalence of these disorders in Hamadan was less than the prevalence in other cities of Iran. These findings can be helpful for large-scale planning for children and adolescents.

## Introduction

Ten to twenty percent of children and adolescents influenced by mental and psychological problems around the globe every year^1^. Child and adolescent psychiatric disorders could have adverse effects on individuals, families, and communities ^2^. Psychiatric disorders not only cause personal and family problems but also are determining risk factors for substance abuse and criminality in adolescents which can predict negative consequences in adulthood as well^3^.

The prevalence of psychiatric disorders in 1998 was 12.5% and 11.1% in 2013 to 2014^4^. A recent systematic review and meta-analysis of the prevalence of psychiatric disorders in adolescents in 27 countries showed an overall prevalence of psychiatric disorders of 13.4%^5^. In another study, the prevalence of psychiatric disorders was 10.l%^6^. In Iran, approximately 17.9% of 6-11-yr-old children in Tehran were suffered from psychiatric disorders^7^. In Ardabil Province of Iran, hyperactivity, oppositional defiant, and separation anxiety disorder had the highest prevalence, in contrast, psychosis, autism and panic disorders had the lowest prevalence^8^. The prevalence of psychiatric disorder in males and females was 11.30% and 9.76%, respectively^9^.

There was little change in the overall prevalence of mental disorders between 1998 and 2014 implying that new innovations in research, policy, and practice are needed to successfully address the major public health problem posed by a child and adolescent mental disorders in the community^10^. The failure to consider mental health problems in children and adolescents is a public health problem with wide-reaching consequences because a substantial proportion of mental health problems in adults originates from their early childhood which effects go beyond childhood and adolescence^1^.

Studies on the prevalence of psychiatric disorders in children and adolescents in different parts of the world provide a variety of the different reports and methodological weaknesses in epidemiological studies make it difficult to conclude many of these studies. The results of these studies are typically obtained using parental rating criteria, non-standard methods, small samples, and are often based on available samples, to use systematic methods.

We focused on the prevalence of psychiatric disorders with different methodology in children and adolescents aged 6 to 18 yr in Hamadan Province, west of Iran.

## Methods

This research is a part of a national project conducted in the urban and rural areas of Iran, called “The Iranian Children and Adolescents' Psychiatric Disorders Study (IRCAP)”. The protocol and methodology of IRCAP study are fully described^11^. For evaluating psychiatric disorders in children and adolescents aged 6 to 18 yr, we used a semi-structured interview K-SADS-PL. Overall, 1020 (529 girls and 494 boys) children were sampled through Multistage cluster randomization. In addition to the main city, the rural population were randomly selected (using cluster sampling); in the next step, the blocks were selected randomly according to the postal code. We conducted this cross-sectional study between July 2016 and May 2017 in Hamadan Province.

The inclusion criteria stipulated that children and adolescents should be between 6 and 18 yr of age and be identified as Iranian population. Participants were excluded if the child or adolescent or their parents had restriction that forbidden their ability to sufficiently complete the questionnaire, such as sever developmental or psychosis or learning disabilities, or inability to read and speak Farsi. The interviews were performed by specialist clinical psychologists using the software.

The national institute for medical research development (NIMAD) supported this study (the ethics code of IR.NIMAD.REC.1395.001). Written consent was obtained from each subject. Other clinical and demographic data were also obtained from each individual.

### Evaluation of Psychiatric Disorders

Psychiatric disorders in children and adolescents were evaluated using the Schedule for Affective Disorders and Schizophrenia for School-Age Children/Present and Lifetime Version (KSADS- PL) based on mother/main caregiver report. KSADS- PL is a semi-structured psychiatric interview that helps in diagnosis of psychiatric disorders in five categories:

1. “Affective disorders (depressive disorders [major depression, dysthymia] and mania, hypomania);
Psychotic disorders;
2. Anxiety disorders (social phobia/ agoraphobia/ specific phobia/ obsessive-compulsive disorder/ separation anxiety disorder/ generalized anxiety disorder/ panic disorder/ posttraumatic stress disorder);
3. Disruptive behavioral disorders (ADHD/conduct disorder/oppositional defiant disorder).
4. Substance abuse, tic disorders, eating disorders, and elimination disorders (enuresis/encopresis)”12. The interview opens with questions about basic demographics. Health and developmental history data should be obtained as this information may be helpful in making differential diagnoses12.

The test-retest reliability and inter-rater reliability of the Persian version of K-SADS-PL were 0.81 and 0.69, respectively in which the sensitivity and specificity of the Persian version of K-SADS were high. The K-SADS-PL was used to diagnose ADHD and its psychiatric comorbidities. We considered all the lifespan related psychiatric diagnoses^13^. An acceptable interrater agreement for K-SADS has been reported (kappa coefficients 0.90 to 0.94) ^14^.

### Statistical Analysis

Data were analyzed using SPSS ver. 22 (Chicago, IL, USA). To investigate the relationship between scores of the K- SADS questionnaire and the demographic factors, we used descriptive analysis and 95% confidence interval. We used the multivariable logistic regression to predict the Odds Ratios (ORs)

## Results

The prevalence of psychiatric disorders in boys was higher than girls (12.6% and 4.9%, respectively). Among three age groups, the psychiatric disorders were most prevalent in 6-9 yr old age group (11.0%). The prevalence of psychiatric disorders in rural areas was higher than in urban areas (10.6% and 8.3%, respectively). The prevalence of psychiatric disorders based on the demographic variables such as father’s and mother’s education (MSc or higher), father’s (unemployed) and mother’s job (unemployed or housewife) were more than other variables (Table 1).

**Table 1:** Frequency of Demographic Variables in Children and Adolescents (6-18) of Hamadan province and Prevalence of Psychiatric Disorders in Terms of these Variables

**Variables**	**Total** **n=1020**		**With disorder, n=88**	**Prevalence CI (95%)**
	**Number**	**Percent**	**Number**	
Sex				
Boy	494	48.4	62	12.55(9.91, 15.76 )
Girl	529	51.9	26	4.91 (3.37, 7.10)
Age (yr)				
6-9	346	33.9	38	10.98 (1.78, 5.60)
10-14	335	32.8	24	7.16 (4.86, 10.43)
15-18	339	33.2	26	7.67 (5.29, 11.00)
Place of residence		
Urban	869	85.2	72	8.28 (6.64, 10.31)
Rural	151	14.8	16	10.59 (6.63, 16.52)
Father’s education			
Illiterate	42	4.2	3	7.14 (2.46, 19.01)
Primary school	217	21.8	20	9.21 (6.05, 13.81)
Guidance & high school	261	26.2	27	10.34 (7.20, 14.63)
Diploma	227	22.8	17	7.48 (4.73, 11.67)
Bachelor	185	18.6	10	5.41 (2.97, 9.67)
MSc or higher	65	6.5	9	13.85 (7.46, 24.27)
Mother’s education			
Illiterate	27	2.7	2	7.41 (2.06, 23.37)
Primary school	253	24.9	22	8.69 (5.82, 12.82)
Guidance & high school	244	24.0	25	10.25 (7.04, 14.69)
Diploma	294	28.9	22	7.48 (4.99, 11.07)
Bachelor	179	17.6	13	7.26 (4.29, 12.02)
MSc or higher	20	2.0	3	15.00 (5.24, 36.04)
Father’s job				
Public sector	307	30.8	21	6.84 (4.52, 10.23)
Private sector	661	66.4	61	9.23 (7.25, 11.68)
Unemployed	28	2.8	4	14.28 (5.70, 31.49)
Mother’s job				
Public sector	86	8.5	5	5.81 (2.51, 12.89)
Private sector	23	2.3	2	8.69 (2.42, 26.8)
Unemployed	908	89.3	80	8.81 (7.14, 10.83)

The odds ratio of gender (female): (OR=0.36; 95% CI: 0.22, 0.59) (*P*=0.001) and age (10-14): (OR=056; 95% CI: 0.32, 0.98) were statistically significant (*P*=0.049) (Table 2).

**Table 2:** Odds Ratios (95% CI) for total psychiatric disorder in term of demographic variables

**Variables**	**Crude OR ** **(CI 95%)**	***P*** ** value**	**Adjusted OR** **(CI 95%)**	***P*** ** value**
Sex				
Male	1.00		1.00	
Female	0.36 (0.22, 0.58)	0.001	0.36 (0.22, 0.59)	0.001
Age group (yr)				
6-9	1.00		1.00	
10-14	0.62 (0.36, 1.06)	0.091	0.56 (0.32, 0.98)	0.049
15-18	0.67 (0.39, 1.13)	0.145	0.65 (0.37, 1.13)	0.127
Locus of life				
Urban	1.00		1.00	
Rural	1.31 (0.74, 2.32)	0.352	1.16 (0.61, 2.21)	0.652
Father’s education				
Illiterate	1.00		1.00	
primary school	1.32 (0.37, 4.65)	0.666	1.28 (0.32, 5.01)	0.723
High school	1.50 (0.43, 5.18)	0.522	1.28 (0.31, 5.22)	0.728
Diploma	1.05 (0.29, 3.76)	0.937	1.11 (0.25, 4.90)	0.891
Bachelor	0.74(0.19, 2.83)	0.663	1.07 (0.20, 5.66)	0.934
MSc or higher	2.08 (0.53, 8.21)	0.292	3.04 (0.51, 18.35)	0.225
Mother’s education				
Illiterate	1.00		1.00	
primary school	1.19 (0.26, 5.36)	0.820	0.94 (0.18, 4.77)	0.939
High school	1.42 (0.32, 6.38)	0.642	1.32 (0.25, 7.01)	0.747
Diploma	1.01 (0.22, 4.55)	0.989	0.95 (0.17, 5.36)	0.958
Bachelor	0.98 (0.21, 4.60)	0.978	0.95 (0.15, 6.0)3	0.953
MSc or higher	2.20 (0.33, 14.63)	0.413	1.99 (0.19, 20.39)	0.561
Father’s job				
Public sector	1.00		1.00	
Private sector	1.38 (0.83, 2.32)	0.216	1.45 (0.68, 3.06)	0.334
unemployed	2.27 (0.72, 7.15)	0.161	2.21 (0.58, 8.41)	0.247
Mother's job				
Public sector	1.00		1.00	
Private sector	1.54 (0.28, 8.52)	0.619	2.21 (0.32, 15.03)	0.419
Unemployed (Housewife)	1.56 (0.62, 3.97)	0.346	1.83 (0.56, 5.96)	0.312

**Figure 1:**
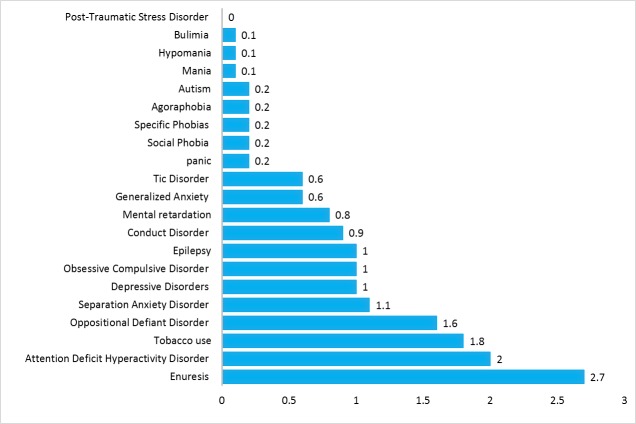
The prevalence of psychiatric disorders in children and adolescents in Hamadan Province

**Figure 2:**
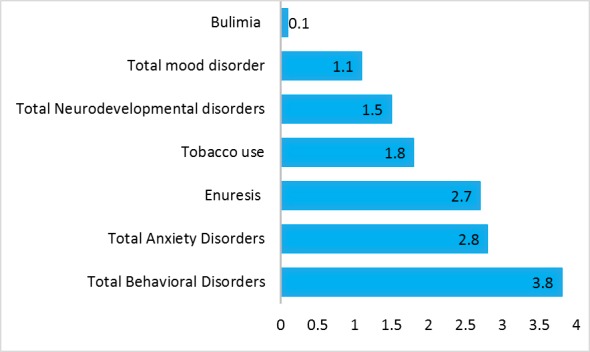
The prevalence of psychiatric disorders in children and adolescents in Hamadan Province

The prevalence of total psychiatric disorders in Hamadan Province was 8.6%. The prevalence of behavioral disorders was 3.8% with attention deficit hyperactivity disorder (ADHD) as the most prevalent one with a prevalence equal to 2.0%. The prevalence of anxiety disorders was 2.8% in which the highest prevalence belonged to separation anxiety disorder (SAD) (1.1%). The prevalence of neurodevelopment disorders was 1.5% with the highest prevalence of 1% observed in epilepsy. The prevalence of mood disorders was 1.1% with depressive disorder as the most prevalent one (1.0%). The prevalence of enuresis and tobacco use was 2.7% and 1.8%, respectively (Table 3, Figure 1 and Figure 2).

**Table 3:** The Prevalence of Psychiatric Disorders in the Hamedan province children and adolescents (6-18)

**Psychiatric Disorders**	**Number**	**Prevalence CI** **(95%)**
Mood disorders		
Depressive disorders	10	0.98 (0.53, 1.79)
Mania	1	0.10 (0.02, 0.56)
Hypomania	1	0.10 (0.02, 0.56)
Total mood disorder	11	1.07 (0.60, 1.92)
Anxiety disorders		
Panic	2	0.20 (0.06, 0.72)
Separation anxiety disorder	11	1.07 (0.60, 1.92)
Social phobia	2	0.20 (0.06, 0.72)
Specific phobias	2	0.20 (0.06, 0.72)
Agoraphobia	2	0.20 (0.06, 0.72)
Generalized anxiety	6	0.59 (0.27, 1.28)
Obsessive compulsive disorder	10	0.98 (0.53, 1.79)
Post-traumatic stress disorder	0	0.00 (0.00, 0.00)
Total anxiety disorders	29	2.84 (1.98, 4.05)
Behavioral disorders		
Attention deficit hyperactivity disorder	20	1.96 (1.27, 3.01)
Oppositional defiant disorder	16	1.57 (0.97, 2.53)
Conduct disorder	9	0.88 (0.46, 1.67)
Tic disorder	6	0.59 (0.27, 1.28)
Total behavioral disorders	39	3.82 (2.81, 5.18)
Neurodevelopmental disorders		
Mental retardation	8	0.78 (0.40, 1.53)
Autism	2	1.96 (0.06, 0.72)
Epilepsy	10	0.98 (0.53, 1.79)
Total	15	1.47 (0.89, 2.41)
Tobacco use	18	1.76 (1.12, 2.77)
Enuresis	28	2.74 (1.91, 3.94)
Bulimia	1	0.10 (0.02, 0.56)
Total psychiatric disorders	88	8.62 (7.06, 10.51)

The most common comorbidities were in anxiety and mood disorders 5 (50.0%), mood and behavioral disorders 4 (40.0%), developmental and behavioral disorders 5 (33.3%), behavioral and substance abuse disorders 5 (27.8%), anxiety and behavioral disorders 6 (20.7) (Table 4).

**Table 4:** Comorbidity disorders according to the type of psychiatric disorder in Hamadan Province

**Main disorder**	**Mood** **Disorders**	**Anxiety** ** disorders**	**Behavioral** ** Disorders**	**Neurodevelopmental** **disorders**	**Substance abuse** **disorders**	**Elimination** **Disorders**	**Eating** **Disorder**
Mood Disorders	11	5	4	1	1	0	1
Anxiety Disorders	5	29	6	1	2	4	0
Behavioral Disorders	4	6	39	5	5	3	1
Neurodevelopmental disorders	1	1	5	15	0	2	0
Substance abuse disorders	1	2	5	0	18	0	0
Elimination Disorders	0	4	3	2	0	28	0

## Discussion

This study as a first one, was carried out in Hamadan Province with a large sample selected from the urban and rural areas to determine the prevalence rates of psychiatric disorders in children and adolescents in the age group of 6-18 yr old. The overall prevalence of psychiatric disorders in the studied age group (6-18) was 8.6% (95% CI: 7.06, 10.51). This finding is approximately consisted with previous study conducted in Iran in this age group (10.51%)^9^. The overall prevalence of psychiatric disorders in 1998 was 12.5% (95% CI: 11.4, 13.7) and 11.1% (95% CI: 10.1, 12.2) in 2013 to 2014^4^.

Some distinct differences were found among groups in terms of the prevalence of disorders based on the demographic variables, with higher prevalence in three age groups of 6-9, 10-14 and 15-18 yr old. The prevalence of psychiatric disorders in boys was higher than girls and the psychiatric disorders were most prevalent in the 6-9 yr old subjects which are in line with previous findings ^9,15-18^. The prevalence of psychiatric disorders was higher in rural areas compared to urban areas, not consistent with the previous findings^19, 20^. In the United States, no association was observed between urban areas and the prevalence of major depression in adolescents. In addition, no significant differences were found in the prevalence of major depression or serious mental illness in adults between large metropolitan areas and rural areas, while the prevalence of both was slightly higher in two intermediate urban areas compared to large metropolitan areas. Contrary to expectations, the prevalence of mental disorders was not higher in most of the urban compared to rural areas^21^.

The prevalence of psychiatric disorders based on the demographic variables such as father’s and mother’s education (MSc or higher), father’s (unemployed) and mother’s job (unemployed or housewife) were more than other variables which are in line with the results of the other studies^22,23^.

Another important finding of this study was the higher prevalence of psychiatric disorders in parents with higher education, in line with other study^22^. The odds ratios of psychiatric disorders were higher in girls aged between 10-14 yr old. This finding is also consistent with previous study^23^. Total behavioral disorder was 3.8% with attention deficit hyperactivity disorder (ADHD) as the most prevalent one (2%) which is consistent with the results of other studies^3^, ^23^. The prevalence of total neurodevelopment disorder was 1.5% with the highest prevalence of 1% observed in epilepsy. The prevalence of total mood disorder was 1.1% with depressive disorder as the most prevalent one (1%). The prevalence of enuresis and tobacco use was 2.7% and 1.8, respectively, this prevalence of tobacco use was less than other finding^24^. ADHD, ODD, enuresis, and SAD disorders had the highest prevalence in the studied sample. In contrast, psychosis, autism, and panic disorders had the lowest prevalence. This finding was concordant with other study conducted in province of Iran ^8.^ Conduct disorders 6.6% (7.1% for child sample and 6.0% for adolescent sample) and anxiety disorders 5.0% (5.9% for child sample and 6.0% for adolescent sample) were the most common groups of disorders and the risk factors are related to child characteristic (gender, poor general health, and stressful life experiences)^25^. The overall prevalence of psychiatric disorders in children and adolescents was 13.4% (CI 95% 11.3-15.9). The worldwide prevalence of anxiety, depressive and attention-deficit hyperactivity disorders were 6.5%, 2.6%, and 3.4%, respectively validated by our findings^5^.

In our study, the most common comorbidities were anxiety and mood disorders, mood and behavioral disorders, developmental and behavioral disorders, behavioral and substance abuse disorders and anxiety and behavioral disorders. More comorbid disorders; from anxiety disorder to conduct disorder and emotional disorders (depression or anxiety have highest comorbidity)^25^. The most common observed combinations were any mood disorder with any ADHD/hyperactivity disorder, any anxiety disorder with any mood disorder, seen in 16 subjects (21.9%), any ADHD/hyperactivity disorder with any conduct/oppositional disorder, and any anxiety disorder with any ADHD/ hyperactivity disorder ^3^. These findings are consistent with the findings of our study.

Some of the advantages of this study were that it is a population-based study, with face-to-face interviews, involving a large sample of children and adolescents. We used an international instrument, designed to generate diagnoses of psychiatric disorders in children and adolescents, validated for using in Iran applied by trained psychologists. The limitations of our study were based only on information obtained from the parents, children, and adolescents, because the KSADS- PL was not administered to children and adolescent teachers. Teachers’ reports could reveal some other symptoms not recognized by parents, contributing to a more accurate diagnosis of the psychiatric disorders.

## Conclusion

The prevalence of these disorders (with a small difference) in Hamadan is less than the prevalence in other cities of Iran. These findings can be helpful for large-scale planning for children and adolescents.

## Acknowledgements

We would like to thank all the interviewers involved in the study in Hamadan Province. We would also like to thank all the families of the participants for their cooperation.

## Conflict of interest statement

The authors declare that there is no conflict of interests.

## Funding

This study was supported by National Institution for Medical Research Development Islamic Republic of Iran (NIMAD) [Grant No. 940906]. Psychiatry and Psychology Research Center, Tehran University of Medical Sciences, and Research Center for Behavioral Disorders and Substances Abuse, Hamadan University of Medical Sciences Hamadan, Iran, involved in this survey.

## Highlights

- The prevalence of total psychiatric disorder was 8.6%.
- The psychiatric disorders were most prevalent in 6-9 yr old age group (11%).
- The highest prevalence was in behavioral disorders (attention deficit hyperactivity disorder) and the least prevalence was in mood disorder (depressive disorder).
